# Bleeding ‘downhill’ esophageal varices associated with benign superior vena cava obstruction: case report and literature review

**DOI:** 10.1186/s12876-016-0548-7

**Published:** 2016-10-24

**Authors:** Michael Loudin, Sharon Anderson, Barry Schlansky

**Affiliations:** 1 Department of Medicine, Division of Gastroenterology & Hepatology, Oregon Health & Science University, 3181 SW Sam Jackson Park Road, Portland, OR L-461 USA; 2 Department of Medicine, Division of Nephrology, Oregon Health & Science University, 3181 SW Sam Jackson Park Road, Portland, OR USA

**Keywords:** Case report, Esophagus, Bleeding varices, Vascular obstruction, Superior vena cava, Proximal esophageal varices

## Abstract

**Background:**

Proximal or ‘downhill’ esophageal varices are a rare cause of upper gastrointestinal hemorrhage. Unlike the much more common distal esophageal varices, which are most commonly a result of portal hypertension, downhill esophageal varices result from vascular obstruction of the superior vena cava (SVC). While SVC obstruction is most commonly secondary to malignant causes, our review of the literature suggests that benign causes of SVC obstruction are the most common cause actual bleeding from downhill varices. Given the alternative pathophysiology of downhill varices, they require a unique approach to management. Variceal band ligation may be used to temporize acute variceal bleeding, and should be applied on the proximal end of the varix. Relief of the underlying SVC obstruction is the cornerstone of definitive treatment of downhill varices.

**Case presentation:**

A young woman with a benign superior vena cava stenosis due to a tunneled internal jugular vein dialysis catheter presented with hematemesis and melena. Urgent upper endoscopy revealed multiple ‘downhill’ esophageal varices with stigmata of recent hemorrhage. As there was no active bleeding, no endoscopic intervention was performed. CT angiography demonstrated stenosis of the SVC surrounding the distal tip of her indwelling hemodialysis catheter. The patient underwent balloon angioplasty of the stenotic SVC segment with resolution of her bleeding and clinical stabilization.

**Conclusion:**

Downhill esophageal varices are a distinct entity from the more common distal esophageal varices. Endoscopic therapies have a role in temporizing active variceal bleeding, but relief of the underlying SVC obstruction is the cornerstone of treatment and should be pursued as rapidly as possible. It is unknown why benign, as opposed to malignant, causes of SVC obstruction result in bleeding from downhill varices at such a high rate, despite being a less common etiology of SVC obstruction.

## Background

‘Downhill’ esophageal varices are an uncommon etiology of gastrointestinal bleeding, estimated to account for approximately 0.1 % of all cases of variceal hemorrhage [1, 2]. The most common reported cause of SVC compression is from mediastinal malignancy such as thymoma, lymphoma or lung cancer, accounting for approximately 60 % of cases [3]. Although bleeding ‘downhill’ varices are rare, non-bleeding varices have been reported to occur in 30 % of patients with benign or malignant SVC obstruction undergoing screening upper endoscopy [1]. SVC obstruction diverts venous return from the head and upper torso through collaterals such as the azygous or innominate veins to bypass the obstruction. The proximal and mid esophageal veins drain into the azygous and innominate veins, and the increased pressure and collateralization result in the development of esophageal varices supplied from the superior aspect of the esophagus and extending distally [4].

We performed a literature search within the MEDLINE and SCOPUS databases using the search strings “proximal varices” and “downhill varices” to identify case reports or studies involving “downhill” varices. Interestingly, while malignancy is described as the most common underlying etiology of SVC obstruction (60 %), based on a review of the available literature, malignancy is the reported etiology for only 14 % of SVC obstruction resulting in downhill variceal bleeding (Table 1). The most common etiology of bleeding downhill varices is a complication related to a venous catheter (27 %), with our patient representing the 10^th^ reported case in the literature. Other benign etiologies of SVC obstruction such as mediastinal fibrosis, behcet’s, goiter, thrombus or post-surgical complications account for the majority of the remaining reported cases of benign obstruction resulting in bleeding. Some theories have been proposed regarding why downhill varices bleed less than distal esophageal varices. These include less exposure to gastric acid, the fact that proximal esophageal varices are submucosal as opposed to the more superficially located distal esophageal varices which are found in the subepithelial venous plexus, and that patients with proximal esophageal varices generally lack the coagulopathy associated with liver dysfunction commonly found in patients with distal esophageal varices [5]. However no explanation is available as to why benign etiologies of SVC obstruction leading to bleeding downhill varices are reported in the literature at a much higher frequency than those associated with malignant obstruction, despite malignancy being the predominant cause of SVC obstruction in the general population.

**Table 1:** Etiologies and therapies of proximal esophageal variceal hemorrhage in case series

Citation	Etiology of proximal esophageal varices	Number of patients	Treatment (n)
Nayudu et al. 2013, USA [8], Vorlop et al 2008, USA [9], Froilan et al. 2008, Spain [6], Hussein et al. 2008, USA [10], Greenwell et al. 2007, USA [11], Blam et al. 2002, USA [12], Pop et al. 1998, USA [13], Gopaluni at al. 2009, UK [14]	Central venous catheter	9	Angioplasty (2), angioplasty and superior vena cava stenting (2), sclerotherapy and superior vena cava stenting (1), surgical vascular reconstruction (1), removal of central venous catheter (1), supportive care (1)
Pashankar et al. 1999, Canada [15], Mikkelson at al. 1963, USA [16], Savoy et al. 2004, USA [17], Sundermann et al. 1960, Germany [18], Johnson et al. 1978 Canada [19]	Thoracic Malignancy	5	Sclerotherapy (1), band ligation and superior vena cava stenting (1), surgical resection of cancer (1), supportive care (1), not reported (1)
Yasar et al. 2015, Turkey [20], Basaranoglu et al. 1999, Turkey [21], Glanz et al. 1982, USA [22], Pugliese 1973, USA [23], Snodgrass et al. 1961, USA [24]	Mediastinal Fibrosis	5	Conservative (2), steroids (1), Sengstaken-Blakemore tube (1), not reported (1)
Papazian et al. 1983, France [25], Palmer et al. 1952, USA [26]	Superior vena cava obstruction (not otherwise specified)	4	Conservative (3), not reported (1)
Ibis et al. 2007, Turkey [27], Fleig et al. 1982, USA [28], Kelly et al. 1982, USA [29]	Thyroid goiter	3	Band ligation (1), Sengstaken-Blakemore tube (1), surgical resection of thyroid goiter (1)
Tavakkoli at al. 2006, Iran [30], Ichikawa et al. 1991, Japan [31]	Behcet’s syndrome	2	Band ligation (1), supportive care (1)
Calderwood et al. 2008, USA [32], Maton et al. 1985, USA [33]	Upper extremity DVT	2	Band ligation, angioplasty, and superior vena cava stenting (1), conservative (1)
Tincani et al. 1998, Italy [34]	Cirrhosis	1	Diagnosis at autopsy
Malloy et al. 2013, USA [35]	Post Fontan cardiac surgery	1	Angioplasty and superior vena cava stenting
Tsokos et al. 1998, Germany [36]	Post thyroidectomy	1	Sclerotherapy
Areia et al. 2006, Portugal [2]	Pulmonary hypertension	1	Supportive care
Pillai et al. 2013, USA [4]	Hemodialysis reliable outflow (HeRO) graft associated	1	Band ligation
Martorell et al. 1955, Spain [37]	Ligation of SVC	1	Not reported

*USA* United States of America, *SVC* superior vena cava, *DVT* deep vein thrombosis

The treatment of bleeding ‘downhill’ esophageal varices involves a multidisciplinary team including thoracic or vascular surgery, interventional radiology, and the endoscopist. When possible, correction of the underlying cause of SVC obstruction is the cornerstone of management, and may involve the angiographic dilation of the narrowed SVC segment, surgical reconstruction or resection of the involved SVC, or cancer therapies such as chemotherapy or external beam radiation [6, 7]. Endoscopic therapy with variceal band ligation or sclerotherapy (at the proximal end of the varix from which blood flow is supplied) or balloon tamponade can be attempted when bleeding is severe to temporize bleeding prior to definitive therapy. Endoscopic approaches are technically limited by the proximity of the varices to the larynx and may be painful due to the somatic innervation of the proximal esophagus.

In this paper we report the 10^th^ case of bleeding downhill varices secondary to complications from a central venous catheter, confirming this as the most commonly reported underlying etiology of bleeding downhill varices. It remains uncertain why benign, as opposed to malignant, causes of SVC obstruction result in bleeding from downhill varices at such a high rate.

## Case presentation

**Fig. 1:**
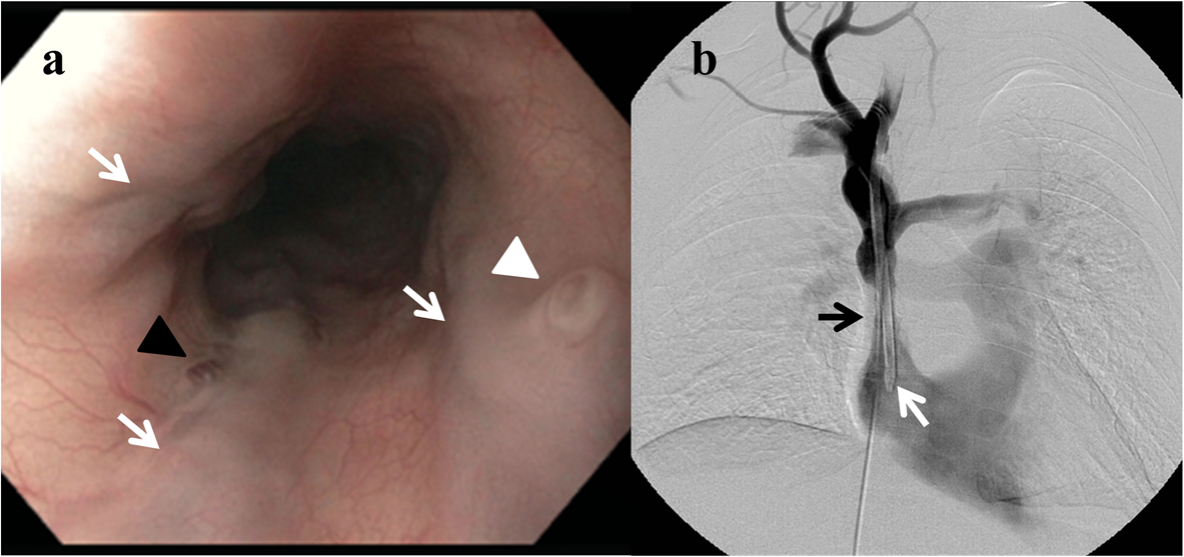
**a** Esophagogastroduodenoscopy in a patient with superior vena cava obstruction demonstrating varices in the proximal esophagus (white arrows), with overlying red wales (black arrowhead) and a fibrin plug (‘nipple sign’) (white arrowhead), indicating recent hemorrhage. **b** Venography of the superior vena cava showing a tunneled dialysis catheter (white arrow) with an adjacent superior vena cava stenosis (black arrow)

A 22 year-old woman presented with acute hematemesis, tachycardia, and hypotension after 3 days of melenic stools. Her only medical history was end-stage kidney disease due to Henoch-Schönlein purpura, and she underwent chronic hemodialysis using a tunneled right internal jugular venous catheter due to prior complications with her right arm fistula. Her current hemodialysis catheter had been in place for approximately 14 months. She had no history of prior liver disease or gastrointestinal bleeding and denied NSAID use. Physical exam was notable for facial edema and erythema (plethora), abdominal and chest wall varices, and tachycardia, without stigmata of chronic liver disease (ascites, splenomegaly, palmar erythema, or spider telangiectasias). Laboratory evaluation revealed an acute anemia (hemoglobin 4.65 mmol/L) with normal platelets, liver function, and coagulation studies. Upper endoscopy was urgently pursued and revealed three columns of large varices in the proximal esophagus with stigmata of recent hemorrhage (Fig. 1a) and a normal distal esophagus, stomach, and duodenum. CT angiogram showed a stenosis in the superior vena cava adjacent to the distal aspect of her hemodialysis catheter with a dilated azygous vein bypassing the stenotic SVC segment to supply the proximal esophageal varices in a retrograde direction.

The patient experienced a second episode of hematemesis and urgently underwent balloon dilation of the stenotic SVC segment under angiography (Fig. 1b). She had no further episodes of gastrointestinal bleeding and her vital signs normalized immediately after the procedure. She was discharged shortly thereafter and underwent a repeat balloon dilation of the stenotic SVC segment 1 week after discharge. She did not experience recurrent gastrointestinal hemorrhage over a 12-month follow up period after her hospitalization.

## Conclusions

Providers should be vigilant for bleeding “downhill” varices in patients with upper gastrointestinal bleeding and clinical evidence of SVC obstruction because the pathophysiology of this disorder mandates a unique management compared to esophageal varices occurring in the usual setting of portal hypertension and cirrhosis. Though data is lacking, traditional medical management would be unlikely to be of benefit in this population. Octreotide, as a splanchnic dilator would not decrease the pressure in “downhill” varices as they do not communicate directly with the portal system. Proton pump inhibitors would be unlikely to play a role as the upper esophagus is less likely to be influenced by gastric pH. The current literature only provides guidance for therapy by means of case reports and while firm recommendations cannot be made as to ideal therapy in this patient population, several methods of temporization seem to have been successful in halting bleeding until definitive decompression of the affected vessels can be performed. Further investigation is required to determine why benign, as opposed to malignant, causes of SVC obstruction result in bleeding from downhill varices at such a high rate, despite being a less common etiology of SVC obstruction in the general population.

## Abbreviations

DVT: Deep vein thrombosis
SVC: Superior vena cava
USA: United States of America
